# Elevated Indoxyl Sulfate Levels Correlate with Increased Aortic Stiffness in Patients Undergoing Kidney Transplantation

**DOI:** 10.3390/toxins18020071

**Published:** 2026-01-30

**Authors:** Hsiao-Hui Yang, Chin-Hung Liu, Yen-Cheng Chen, Bang-Gee Hsu

**Affiliations:** 1 Division of General Surgery, Department of Surgery, Hualien Tzu Chi Hospital, Buddhist Tzu Chi Medical Foundation, Hualien 97004, Taiwan; 2 School of Medicine, Tzu Chi University, Hualien 97004, Taiwan; 3 Institute of Medical Science, Tzu Chi University, Hualien 97004, Taiwan; 4 Graduate Institute of Clinical Pharmacy, School of Medicine, Tzu Chi University, Hualien 97004, Taiwan; 5 School of Pharmacy, Tzu Chi University, Hualien 97004, Taiwan; 6 Division of Nephrology, Hualien Tzu Chi Hospital, Buddhist Tzu Chi Medical Foundation, Hualien 97004, Taiwan

**Keywords:** indoxyl sulfate, aortic stiffness, carotid–femoral pulse wave velocity, kidney transplantation, uremic toxins

## Abstract

Although kidney transplantation (KT) restores renal function, residual uremic toxins, such as indoxyl sulfate (IS), may persist and contribute to vascular remodeling and aging. Aortic stiffness, reflected by carotid–femoral pulse wave velocity (cfPWV), is a strong predictor of cardiovascular events. This study enrolled KT recipients to examine the association of circulating IS with aortic stiffness. Using the SphygmoCor system, we assessed aortic stiffness, which was defined as cfPWV > 10 m/s. Serum IS concentrations were measured by liquid chromatography–tandem mass spectrometry. Of 94 KT recipients, 26 (27.7%) met the criteria for aortic stiffness. Compared with patients without aortic stiffness, those with aortic stiffness were older (*p* = 0.017) and had significantly higher systolic blood pressure (*p* = 0.011) and fasting glucose levels (*p* = 0.002), a higher prevalence of diabetes (*p* = 0.043), and higher IS levels (*p* = 0.002). According to multivariable logistic regression, serum IS remained independently associated with aortic stiffness (*p* = 0.017). According to stepwise linear regression, log-transformed IS further showed a positive correlation with cfPWV (*p* = 0.016). Serum IS remained an independent determinant of aortic stiffness in KT recipients, highlighting the burden of residual uremic toxins as a contributor to post-transplant vascular aging.

## 1. Introduction

For patients with end-stage kidney disease, kidney transplantation (KT) offers a substantial survival benefit over dialysis, mainly due to reductions in cardiovascular mortality [1]. However, despite advances in perioperative care, tighter metabolic control, and a declining overall burden of cardiovascular disease, cardiovascular events remain the leading cause of death among KT recipients [2,3]. Conventional cardiovascular risk factors, such as hypertension, diabetes, dyslipidemia, and smoking, often persist after transplantation and may be further exacerbated by immunosuppressive therapy, impaired graft function, and post-transplant metabolic disturbances [4,5,6].

In addition to these traditional factors, uremic toxins and gut microbiota-derived metabolites have emerged as important contributors to vascular injury, even in KT recipients who achieve partial recovery of renal function [7]. Aortic stiffness, assessed by carotid–femoral pulse wave velocity (cfPWV), is a well-established non-invasive marker of vascular aging and a strong predictor of cardiovascular mortality [8]. Indoxyl sulfate (IS) is a protein-bound uremic toxin derived from gut microbial metabolism, and it is of particular interest, because it accumulates early in the progression of chronic kidney disease (CKD) and exerts multiple deleterious biological effects [7]. IS induces oxidative stress and inflammation, activates the intrarenal renin–angiotensin–aldosterone system, and promotes tubular injury and fibrosis [9,10]. Experimental evidence further indicates that IS stimulates vascular smooth muscle cell proliferation through mitogen-activated protein kinase signaling [11], thereby contributing to arterial remodeling and the progression of atherosclerotic changes. Elevated IS levels have been associated with vascular calcification, increased arterial stiffness, and adverse cardiovascular outcomes in CKD and end-stage kidney disease [12,13], as well as the development of heart failure in patients on hemodialysis [14].

Despite extensive evidence in nontransplant CKD populations, the cardiovascular impact of IS in KT recipients remains insufficiently understood. KT recipients represent a unique clinical population, in whom residual uremic toxin burden may persist despite improved graft function, and immunosuppressive therapy may further modify toxin-related vascular injury. The correlation between circulating IS levels and aortic stiffness, an early and quantifiable marker of vascular damage, has not been well studied. Therefore, this study investigated the association between serum IS concentrations and cfPWV in KT recipients and evaluated the potential of IS as a biomarker for post-transplant cardiovascular risk.

## 2. Results

Table 1 summarizes the clinical characteristics of 94 KT recipients included in the analysis. Of these, 26 patients (27.7%) met the criteria for aortic stiffness, while 68 patients (72.3%) comprised the control group. Compared with the controls, patients in the aortic stiffness group were significantly older (*p* = 0.017) and had significantly higher systolic blood pressure (SBP, *p* = 0.011), fasting glucose (*p* = 0.002), prevalence of diabetes mellitus (*p* = 0.043), and serum IS levels (*p* = 0.002). There were no significant differences in sex, lipid profile, renal function, donor type, and use of immunosuppressive or lipid-lowering agents between the groups.

According to multivariable logistic regression adjusting for the significant covariates in Table 1 (i.e., diabetes mellitus, age, SBP, fasting glucose, and IS), IS remained the only independent predictor of aortic stiffness [odds ratio 1.320, 95% confidence interval (CI) 1.050–1.659; *p* = 0.017] (Table 2). Variance inflation factors for all predictor variables were <1.5, so no additional collinearity diagnostics were conducted in the multivariable analysis.

Associations between clinical variables and cfPWV were evaluated using simple linear regression and multivariable forward stepwise linear regression analyses. According to simple linear regression, cfPWV was significantly and positively associated with age (*r* = 0.330, *p* = 0.001); SBP (*r* = 0.354, *p* < 0.001); log-transformed IS (log-IS, *r* = 0.350, *p* < 0.001); and log-glucose (*r* = 0.383, *p* < 0.001). Variables with significant univariable associations were subsequently entered into a forward stepwise multivariable model. After adjusting for age, SBP, log-glucose, and log-IS, cfPWV remained independently associated with age (β = 0.192, adjusted R^2^ change = 0.027, *p* = 0.035); SBP (β = 0.251, adjusted R^2^ change = 0.092, *p* = 0.006); log-glucose (β = 0.289, adjusted R^2^ change = 0.137, *p* = 0.002); and log-IS (β = 0.219, adjusted R^2^ change = 0.054, *p* = 0.016) (Table 3).

Log-IS levels were positively correlated with age (*r* = 0.210, *p* = 0.042) and cfPWV (*r* = 0.350, *p* = 0.001) and inversely correlated with high-density lipoprotein cholesterol (HDL-C, *r* = −0.206, *p* = 0.046) and estimated glomerular filtration rate (eGFR, *r* = −0.245, *p* = 0.017) (Table 4).

**Figure 1:**
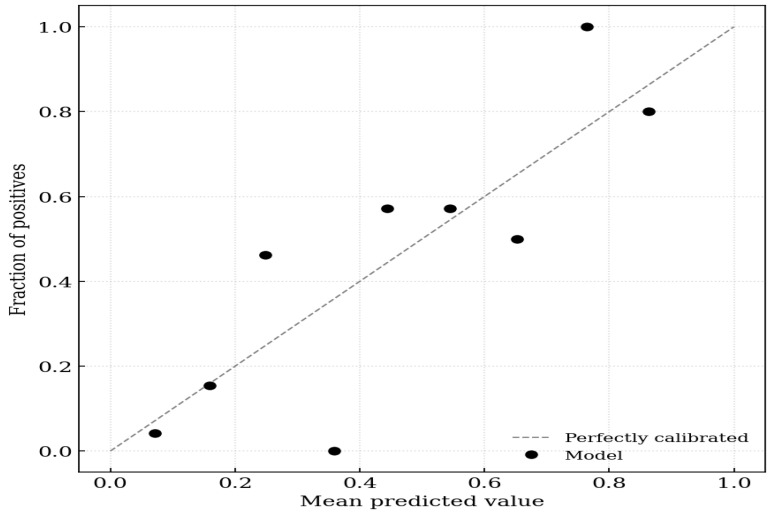
Calibration plot for aortic stiffness. The x-axis represents the mean predicted probability of the outcome, and the y-axis represents the observed fraction of positives. The dashed diagonal line represents perfect calibration (where predicted probability equals observed probability). The solid black circles indicate the model’s performance across 10 groups (deciles) of predicted risk.

On evaluation of calibration, the logistic regression model incorporating IS achieved a Brier score of 0.152, indicating high probabilistic accuracy. The calibration intercept of 0.000 (95% CI −0.657 to 0.657) suggested no systematic bias in the predicted probabilities (calibration-in-the-large). The calibration slope of 1.000 (95% CI 0.516–1.484) indicated ideal discrimination spread without evidence of overfitting or underfitting, because the confidence interval included the value of 1 (Figure 1).

**Figure 2:**
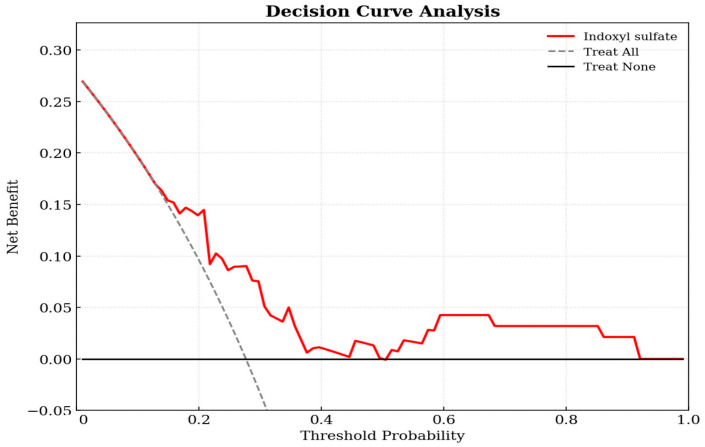
Decision curve analysis of the indoxyl sulfate-based logistic regression model for predicting aortic stiffness in kidney transplant patients. The y-axis measures the net benefit. The x-axis displays the threshold probability. The red solid line represents the net benefit of using the indoxyl sulfate prediction model. The gray dashed line represents the assumption that all patients have aortic stiffness (Treat All), while the black horizontal line represents the assumption that no patients have the condition (Treat None).

Decision curve analysis was utilized to evaluate the clinical utility of the prediction model in the cohort of 94 KT patients. As shown in Figure 2, the decision curve for the IS-based model (red line) was positioned above the two reference lines for treat-all and treat-none across a broad range of threshold probabilities. This indicates that using IS levels to guide clinical interventions yields a higher net benefit than intervening in all patients or none, confirming the model’s clinical value. The decision curve demonstrates that the IS model provides a superior net benefit compared to the default strategies across a wide range of threshold probabilities (approximately 0.2–0.8).

## 3. Discussion

In this study, KT recipients with aortic stiffness were older and demonstrated higher SBP, prevalence of diabetes mellitus, fasting glucose levels, and serum IS levels compared with those in patients without aortic stiffness. Importantly, the positive and independent association of IS levels with cfPWV indicated that IS contributes to vascular stiffness beyond traditional metabolic and hemodynamic risk factors. Decision curve analysis demonstrated superior net clinical benefit over default strategies across threshold probabilities. Taken together, these observations supported the concept that residual uremic toxins, particularly protein-bound solutes, such as IS, remain a clinically meaningful burden even after successful KT and may serve as indicators of post-transplant vascular risk.

Aortic stiffness is an early manifestation of vascular aging, reflecting cumulative structural and functional alterations within the arterial wall. Systemic conditions, such as low-grade inflammation, obesity, and metabolic syndrome, further amplify these processes, reflecting the multifactorial nature of aortic stiffness as an integrative marker of vascular decline [15]. Hyperglycemia, dyslipidemia, and hypertension promote oxidative stress and microvascular injury at arterial bifurcations, triggering endothelial activation, release of chemokines, and sustained inflammation [16]. Together with increased SBP, age-related vascular deterioration, which is characterized by elastin fragmentation, collagen accumulation, extracellular matrix remodeling, and vascular smooth muscle cell proliferation and phenotypic switching, contributes to medial thickening and progressive arterial remodeling [17,18,19]. Disturbances in glucose metabolism further exacerbate arterial stiffening. In fact, even modest elevations in fasting glucose were associated with increased stiffness in nondiabetic populations, and higher cfPWV values were consistently observed in patients with type 2 diabetes than in normoglycemic controls [20,21]. Our findings were consistent with these established mechanisms. Age, SBP, and fasting glucose showed significant associations and remained independent determinants of cfPWV on multivariate analysis, reinforcing the concept that metabolic and hemodynamic stressors continue to shape vascular integrity despite restoration of glomerular filtration after KT.

Beyond these conventional risk factors, IS emerged as an independent correlate of aortic stiffness. IS originates from hepatic sulfation of indole, which is produced by bacterial tryptophan metabolism, and is now recognized as an active uremic toxin rather than a passive marker of impaired renal clearance [7]. A substantial body of experimental evidence demonstrated its direct vascular toxicity by enhancing endothelial adhesion molecule expression, augmenting leukocyte–endothelial interactions, and establishing a persistent proinflammatory vascular environment [7,10]. IS induces oxidative stress through activation of nicotinamide adenine dinucleotide phosphate oxidase and depletion of antioxidant defenses, such as glutathione, leading to reduced nitric oxide bioavailability and impaired endothelium-dependent vasodilation. IS activates mitogen-activated protein kinase signaling and promotes vascular smooth muscle cell proliferation, hypertrophy, and phenotypic switching, which are changes that contribute to medial thickening and extracellular matrix remodeling [9,10,11]. These biological actions closely parallel the key mechanisms of vascular aging, including chronic oxidative stress, endothelial dysfunction, sustained inflammation, and maladaptive extracellular matrix turnover. This mechanistic concordance strengthens the biological plausibility of our observed association between circulating IS levels and increased arterial stiffness.

Clinical studies on populations with CKD have consistently linked elevated IS concentrations with vascular disease, arterial stiffness, and cardiovascular mortality [12,22]. In KT recipients, IS levels may remain clinically relevant despite restoration of glomerular filtration. Huang et al. demonstrated that protein-bound uremic toxins, including IS and p-cresol, remained markedly elevated in KT recipients and were relatively high in those with more advanced stages of CKD, suggesting persistent exposure to gut-derived toxins and their potential role in graft deterioration [23]. In this study, IS levels were inversely correlated with eGFR in KT recipients. IS activates prooxidant, proinflammatory, and prothrombotic mechanisms within endothelial cells, leading to endothelial dysfunction. The resulting impairment promotes arteriosclerosis, disrupts vascular repair, and increases the risk of thrombosis [24]. Ex vivo experiments confirmed that IS selectively disrupts nitric oxide-mediated vasodilation in resistance arteries [25,26]. Taken together, these observations indicated that IS remains biologically active in the post-transplant milieu and may contribute to vascular dysfunction even after partial recovery of renal function.

However, not all findings have been uniform. Liabeuf et al. reported that IS levels decreased rapidly and were not associated with cardiovascular events or mortality during the early post-transplant window (i.e., first year after KT) [27]. These discrepancies may reflect differences in timing, because early IS kinetics largely depend on recovery of tubular secretion rather than long-term vascular toxicity. In addition, immunosuppressive agents, such as corticosteroids and cyclosporine, may transiently mitigate IS-related inflammatory signaling by inhibiting nuclear factor kappa-light-chain-enhancer of activated B cells (NF-κB) pathways, potentially diminishing the vascular effects of IS during the early post-transplantation period [27]. Therefore, current evidence suggests that the vascular consequences of IS in KT recipients likely depend on post-transplant duration, tubular function, and exposure to immunosuppressive agents.

This study had several limitations. First, its cross-sectional and single-center design precluded inference of causality between serum IS levels and aortic stiffness in KT recipients. Longitudinal or interventional studies are required to clarify temporal relationships. Second, the relatively small sample size may have limited statistical power and reduced the generalizability of our findings. Third, we measured total serum IS rather than the free (unbound) fraction. As IS is a protein-bound uremic toxin, total concentrations may not fully reflect biologically active exposure, potentially leading to an underestimation of the strength of the observed association. Fourth, lifestyle factors, such as dietary patterns, physical activity, and medication adherence, were not assessed; these variables may also influence IS production, clearance, and vascular function. These limitations highlight the need for larger, multicenter, and prospective investigations to validate our results and further elucidate the mechanistic role of IS in post-transplant vascular health.

## 4. Conclusions

In conclusion, this study demonstrated that serum IS was independently associated with aortic stiffness in KT recipients and may serve as a potential biomarker for cardiovascular risk stratification after KT. The persistence of IS-related vascular effects despite graft recovery highlighted the importance of addressing residual uremic toxicity as part of comprehensive post-transplant care. Future longitudinal trials are warranted to determine whether reducing IS levels through dietary modulation, microbiota-targeted interventions, and toxin-binding strategies can mitigate vascular stiffness and improve long-term cardiovascular outcomes in this high-risk population.

## 5. Materials and Methods

### 5.1. Patients

This cross-sectional observational study included KT recipients who received routine follow-up care at a medical center in eastern Taiwan from 1 January 2022 to 30 June 2022. Among 110 screened KT recipients, 94 were included. Exclusions were due to arteriovenous fistula/graft (*n* = 3), active infection (*n* = 1), heart failure (*n* = 1), acute rejection (*n* = 1), malignancy (*n* = 3), and refusal to participate (*n* = 7). All participants received a detailed explanation of the study procedures and provided written informed consent prior to enrollment. Demographic characteristics, comorbid conditions, and chronic use of immunosuppressive agents, such as tacrolimus, mycophenolate mofetil, corticosteroids, rapamycin, and cyclosporine, were extracted from electronic medical records. Hypertension was defined as current use of antihypertensive therapy, and diabetes mellitus was identified based on a documented diagnosis or prescription of glucose-lowering medications. The study protocol was approved by the Research Ethics Committee of Hualien Tzu Chi Hospital, Buddhist Tzu Chi Medical Foundation (IRB108-219-A). All study procedures adhered to the principles outlined in the Declaration of Helsinki.

### 5.2. Anthropometric and Biochemical Investigations

Body mass index was calculated as weight in kilograms divided by height in meters squared. Following an overnight fast for approximately 8 h, 5 mL of venous blood was collected from each participant and centrifuged at 3000× *g* for 10 min. Biochemical investigations, including fasting glucose, total cholesterol, triglycerides, HDL-C, low-density lipoprotein cholesterol, blood urea nitrogen, creatinine, calcium, and phosphorus, were performed using an automated analyzer (Siemens Advia 1800, Siemens Healthcare GmbH, Erlangen, Germany). Serum intact parathyroid hormone levels were determined using a commercially available enzyme-linked immunosorbent assay kit (Abcam, Cambridge, MA, USA) [28]. The eGFR was calculated using the CKD Epidemiology Collaboration equation.

### 5.3. Determination of Indoxyl Sulfate Levels by High-Performance Liquid Chromatography–Mass Spectrometry

Serum IS concentrations were quantified using modified high-performance liquid chromatography (HPLC)–tandem mass spectrometry, as previously described [29]. In brief, 100 µL of serum was mixed with 100 µL of 50 mM sodium phosphate dibasic heptahydrate in a 1.5 mL microcentrifuge tube. A deuterated IS analog (d4-IS) served as the internal standard. Protein extraction was performed using Novum^®^ simplified liquid extraction cartridges (Phenomenex, Torrance, CA, USA), and IS was eluted with 1.5 mL of ethyl acetate. The eluate was evaporated under nitrogen and reconstituted in 100 µL of methanol. Chromatographic separation was achieved using a Waters^®^ e2695 HPLC system coupled with an ACQUITY QDa^®^ single-quadrupole mass detector (Waters e2695 Separations Module, Waters Corporation, Milford, MA, USA). A 5 µm, 250 × 4.6 mm, 100-Å column (Phenomenex Luna^®^ C18(2); Phenomenex, Torrance, CA, USA) was maintained at 40 °C, with a flow rate of 0.8 mL/min and an injection volume of 30 µL. The mobile phases consisted of 99.9% double-distilled water (A) and 99.9% HPLC-grade methanol (B), both containing 0.1% formic acid. A linear gradient program was applied: 5% to 70% B over 14 min, followed by a 2 min hold at 70% B, then a return to 50% B over 2 min. IS was detected in negative electrospray ionization mode using a vaporization temperature of 400 °C, capillary voltage of 0.8 kV, and cone voltage of 15 V. Selected ion recording was used for mass-to-charge ratios of 211.9 *m*/*z* for IS and 216.0 m/z for d4-IS. Data acquisition and processing were performed using Empower^®^ 3.0 software (New York, NY, USA), and IS concentrations were calculated based on calibration curves generated from IS standards.

### 5.4. Blood Pressure and Aortic Stiffness Measurements

After a 30 min seated rest, three blood pressure measurements were obtained at 5 min intervals, and the mean value was used for analysis. Aortic stiffness was assessed using cfPWV, which was measured by a cuff-based volumetric displacement device (SphygmoCor XCEL, AtCor Medical, Sydney, NSW, Australia) following standardized operating procedures [28]. All assessments were performed in the morning after participants rested supine for at least 10 min in a quiet temperature-controlled room. The XCEL cuff on the left upper arm measured brachial SBP and diastolic blood pressure by oscillometry and was then reinflated to a subdiastolic level. An upper thigh cuff was used to record femoral volume displacement waveforms, from which the XCEL system (SphygmoCor XCEL 1.30 software) computed cfPWV. Based on the European Society of Cardiology and European Society of Hypertension guidelines, a cfPWV of >10 m/s was classified as indicative of aortic stiffness [30].

### 5.5. Statistical Analysis

All statistical analyses were performed using SPSS software (version 25.0; SPSS Inc., Chicago, IL, USA). Normality was assessed using the Kolmogorov–Smirnov test. Normally distributed variables were reported as mean ± standard deviation and compared using two-tailed independent Student’s *t*-test. Non-normally distributed variables, including triglycerides, fasting glucose, blood urea nitrogen, creatinine, intact parathyroid hormone, and IS, were presented as medians with interquartile ranges and analyzed using the Mann–Whitney U test; these variables were further log_10_-transformed when required for parametric analyses. Categorical variables were presented as frequencies and percentages and compared using the chi-square test. Factors associated with aortic stiffness were first examined using univariable analyses; variables with significant associations were then entered into a multivariable logistic regression model to identify independent predictors. Associations between clinical parameters and cfPWV were explored using simple linear regression, followed by forward stepwise multivariable regression for significant predictors. Relationships between serum log-IS concentrations and clinical parameters were assessed using Spearman’s rank correlation coefficients. We assessed model calibration using predicted–observed risk plots and quantified clinical utility through decision curve analysis. A two-sided *p*-value of <0.05 was considered statistically significant.

## Figures and Tables

**Table 1:** Clinical variables of the 94 kidney transplantation patients with or without aortic stiffness.

Characteristics	All Patients (*n* = 94)	Control Group (*n* = 68)	Aortic Stiffness Group (*n* = 26)	*p*-Value
Age (years)	54.33 ± 11.51	52.59 ± 11.65	58.88 ± 9.97	0.017 *
KT vintage (months)	95.21 ± 57.50	92.94 ± 54.89	101.15 ± 63.11	0.535
Height (cm)	160.69 ± 10.20	159.89 ± 10.49	162.79 ± 9.26	0.220
Body weight (kg)	64.85 ± 14.71	63.55 ± 11.48	68.26 ± 20.85	0.166
Body mass index (kg/m^2^)	24.65 ± 4.59	24.42 ± 4.29	25.25 ± 5.32	0.436
Carotid–femoral PWV (m/s)	9.10 ± 1.94	8.15 ± 1.11	11.58 ± 1.32	<0.001 *
Systolic blood pressure (mmHg)	140.35 ± 17.67	137.50 ± 17.10	147.81 ± 17.27	0.011 *
Diastolic blood pressure (mmHg)	82.93 ± 11.22	82.03 ± 10.67	85.27 ± 12.44	0.212
Total cholesterol (mg/dL)	188.18 ± 47.40	184.94 ± 45.69	196.65 ± 51.60	0.286
Triglyceride (mg/dL)	119.50 (84.50–161.50)	114.00 (82.25–150.00)	138.50 (92.75–187.00)	0.176
HDL-C (mg/dL)	52.64 ± 17.38	52.84 ± 15.85	52.12 ± 21.21	0.858
LDL-C (mg/dL)	105.30 ± 37.19	102.95 ± 30.87	111.46 ± 50.38	0.324
Fasting glucose (mg/dL)	94.50 (88.75–110.00)	92.50 (88.00–99.00)	109.50 (94.00–153.75)	0.002 *
Blood urea nitrogen (mg/dL)	25.00 (17.00–35.25)	23.50 (16.25–32.75)	25.50 (18.75–44.75)	0.228
Creatinine (mg/dL)	1.41 (1.16–2.10)	1.35 (1.15–1.90)	1.80 (1.24–2.25)	0.101
eGFR (mL/min)	49.35 ± 24.11	50.51 ± 22.11	46.31 ± 28.97	0.453
Total calcium (mg/dL)	9.37 ± 0.81	9.35 ± 0.73	9.42 ± 1.01	0.687
Phosphorus (mg/dL)	3.31 ± 0.75	3.27 ± 0.76	3.41 ± 0.73	0.426
Intact parathyroid hormone (pg/mL)	82.10 (52.13–149.85)	82.35 (56.35–146.98)	81.70 (50.08–153.85)	0.543
Indoxyl sulfate (μg/mL)	2.15 (1.42–3.80)	1.87 (1.28–3.29)	3.44 (2.02–4.54)	0.002 *
Female, *n* (%)	49 (52.1)	37 (54.4)	12 (46.2)	0.473
Diabetes, *n* (%)	32 (34.0)	19 (27.9)	13 (50.0)	0.043 *
Hypertension, *n* (%)	37 (39.4)	24 (35.3)	13 (50.0)	0.190
Living donor, *n* (%)	19 (20.2)	15 (22.1)	4 (15.4)	0.471
Steroid use, *n* (%)	82 (87.2)	61 (89.7)	21 (80.8)	0.245
Cyclosporine use, *n* (%)	17 (18.1)	14 (20.6)	3 (11.5)	0.308
Tacrolimus use, *n* (%)	72 (76.6)	50 (73.5)	22 (84.6)	0.256
Mycophenolate mofetil use, *n* (%)	75 (79.8)	56 (82.4)	19 (73.1)	0.316
Rapamycin use, *n* (%)	20 (21.3)	12 (17.6)	8 (30.8)	0.164
Statin use, *n* (%)	38 (40.4)	29 (42.6)	9 (34.6)	0.478
Fibrate use, *n* (%)	18 (19.1)	12 (17.6)	6 (23.1)	0.550

Values for continuous variables are shown as mean ± standard deviation after analysis by Student’s *t*-test; variables not normally distributed are shown as median and interquartile range after analysis by the Mann–Whitney U test; values are presented as number (%) and analysis after analysis by the chi-square test. KT, kidney transplantation; PWV, pulse wave velocity; HDL-cholesterol, high-density lipoprotein cholesterol; LDL-cholesterol, low-density lipoprotein cholesterol; eGFR, estimated glomerular filtration rate. * *p* < 0.05 was considered statistically significant.

**Table 2:** Multivariable logistic regression analysis of the factors correlated to arterial stiffness among 94 kidney transplantation patients.

Clinical Variables	Odds Ratio	95% Confidence Interval	*p*-Value
Indoxyl sulfate, 1 μg/mL	1.320	1.050–1.659	0.017 *
Age, 1 year	1.040	0.987–1.097	0.145
Fasting glucose, 1 mg/dL	1.013	0.999–1.027	0.063
Systolic blood pressure, 1 mmHg	1.028	0.997–1.061	0.081
Diabetes, present	0.938	0.256–3.433	0.923

The multivariable logistic regression model included diabetes mellitus, age, systolic blood pressure, fasting glucose, and indoxyl sulfate and showed significant differences in Table 1. * *p* < 0.05 was considered statistically significant.

**Table 3:** Correlation between carotid–femoral pulse wave velocity levels and clinical variables among 94 kidney transplantation patients.

Clinical Variables	Carotid–Femoral Pulse Wave Velocity (m/s)				
	Univariable Regression		Multivariable Regression		
	*r*	*p*-Value	β	Adjusted R^2^ Change	*p*-Value
Age (years)	0.330	0.001 *	0.192	0.027	0.035 *
KT vintage (months)	0.042	0.684	–	–	–
Body mass index (kg/m^2^)	0.184	0.076	–	–	–
Systolic blood pressure (mmHg)	0.354	<0.001 *	0.251	0.092	0.006 *
Diastolic blood pressure (mmHg)	0.077	0.459	–	–	–
Total cholesterol (mg/dL)	0.069	0.507	–	–	–
Log-Triglyceride (mg/dL)	−0.076	0.468	–	–	–
HDL-C (mg/dL)	−0.138	0.185			
LDL-C (mg/dL)	0.135	0.196	–	–	–
Log-Glucose (mg/dL)	0.383	<0.001 *	0.289	0.137	0.002 *
Log-BUN (mg/dL)	0.131	0.209	–	–	–
Log-Creatinine (mg/dL)	0.118	0.256	–	–	–
eGFR (mL/min)	−0.061	0.558	–	–	–
Total calcium (mg/dL)	−0.001	0.994	–	–	–
Phosphorus (mg/dL)	0.044	0.674	–	–	–
Log-iPTH (pg/mL)	0.041	0.694	–	–	–
Log-IS (μg/mL)	0.350	0.001 *	0.219	0.054	0.016 *

Variables with skewed distributions, including triglycerides, glucose, blood urea nitrogen, creatinine, intact parathyroid hormone, and indoxyl sulfate, were log-transformed before analysis. Associations with cfPWV were first examined using univariable linear regression, followed by multivariable stepwise linear regression including age, systolic blood pressure, log-transformed triglycerides, log-transformed glucose, and log-transformed indoxyl sulfate. KT, kidney transplantation; HDL-cholesterol, high-density lipoprotein cholesterol; LDL-cholesterol, low-density lipoprotein cholesterol; BUN, blood urea nitrogen; eGFR, estimated glomerular filtration rate; iPTH, intact parathyroid hormone; IS, indoxyl sulfate. * *p* < 0.05 was considered statistically significant. An en dash (–) indicates variables that were not statistically significant in univariable analysis and therefore not included in the multivariable model.

**Table 4:** Spearman’s correlation coefficients between log-transformed indoxyl sulfate and clinical variables in 94 kidney transplantation patients.

Variables	Spearman’s Correlation Coefficient	*p*-Value
Age (years)	0.210	0.042 *
Body mass index (kg/m2)	0.047	0.655
Post-KT duration (months)	0.132	0.203
Carotid–femoral PWV (m/s)	0.350	0.001 *
Systolic blood pressure (mmHg)	0.159	0.126
Diastolic blood pressure (mmHg)	0.084	0.421
Total cholesterol (mg/dL)	0.088	0.398
Log-Triglyceride (mg/dL)	0.003	0.980
HDL-C (mg/dL)	−0.206	0.046 *
LDL-C (mg/dL)	0.119	0.254
Log-Glucose (mg/dL)	0.172	0.096
eGFR (mL/min)	−0.245	0.017 *
Total calcium (mg/dL)	0.073	0.482
Phosphorus (mg/dL)	0.091	0.385
Log-iPTH (pg/mL)	0.078	0.457

Data on triglyceride, glucose, iPTH, and indoxyl sulfate levels exhibited a skewed distribution and were therefore log-transformed prior to analysis. Analysis of data was performed using the Spearman correlation analysis. KT, kidney transplantation; PWV, pulse wave velocity; HDL-cholesterol, high-density lipoprotein cholesterol; LDL-cholesterol, low-density lipoprotein cholesterol; eGFR, estimated glomerular filtration rate; iPTH, intact parathyroid hormone. * *p* < 0.05 was considered statistically significant (2-tailed).

## Data Availability

The raw data supporting the conclusions of this article will be made available by the authors on request since there is some consent data involved in this study.

## Abbreviations

The following abbreviations are used in this manuscript:

CKD	Chronic kidney disease
cfPWV	Carotid–femoral pulse wave velocity
CI	Confidence interval
eGFR	Estimated glomerular filtration rate
HDL-C	High-density lipoprotein cholesterol
HPLC	High-performance liquid chromatography
IS	Indoxyl sulfate
KT	Kidney transplantation
NF-κB	Nuclear factor kappa-light-chain-enhancer of activated B cells
SBP	Systolic blood pressure

## References

[1] Strohmaier S., Wallisch C., Kammer M., Geroldinger A., Heinze G., Oberbauer R., Haller M.C. (2022). Survival benefit of first single-organ deceased donor kidney transplantation compared with long-term dialysis across ages in transplant-eligible patients with kidney failure. JAMA Netw. Open.

[2] Wolfe R.A., Ashby V.B., Milford E.L., Ojo A.O., Ettenger R.E., Agodoa L.Y., Held P.J., Port F.K. (1999). Comparison of mortality in all patients on dialysis, patients on dialysis awaiting transplantation, and recipients of a first cadaveric transplant. N. Engl. J. Med..

[3] Beaudrey T., Bedo D., Weschler C., Caillard S., Florens N. (2025). From risk assessment to management: Cardiovascular complications in pre- and post-kidney transplant recipients: A narrative review. Diagnostics.

[4] Vincenti F., Friman S., Scheuermann E., Rostaing L., Jenssen T., Campistol J.M., Uchida K., Pescovitz M.D., Marchetti P., Tuncer M. (2007). Results of an international, randomized trial comparing glucose metabolism disorders and outcome with cyclosporine versus tacrolimus. Am. J. Transplant..

[5] Khoshdel A.R., Carney S.L. (2008). Arterial stiffness in kidney transplant recipients: An overview of methodology and applications. Urol. J..

[6] Devine P.A., Courtney A.E., Maxwell A.P. (2019). Cardiovascular risk in renal transplant recipients. J. Nephrol..

[7] Chen M.C., Kuo C.H., Lin Y.L., Hsu B.G. (2025). Gut-derived uremic toxins and cardiovascular health in chronic kidney disease. Tzu Chi Med. J..

[8] Sequí-Domínguez I., Cavero-Redondo I., Álvarez-Bueno C., Pozuelo-Carrascosa D.P., de Arenas-Arroyo S.N., Martínez-Vizcaíno V. (2020). Accuracy of pulse wave velocity predicting cardiovascular and all-cause mortality: A systematic review and meta-analysis. J. Clin. Med..

[9] Sun C.Y., Chang S.C., Wu M.S. (2012). Uremic toxins induce kidney fibrosis by activating intrarenal renin-angiotensin-aldosterone system associated epithelial-to-mesenchymal transition. PLoS ONE.

[10] Hung S.C., Kuo K.L., Wu C.C., Tarng D.C. (2017). Indoxyl sulfate: A novel cardiovascular risk factor in chronic kidney disease. J. Am. Heart Assoc..

[11] Yamamoto H., Tsuruoka S., Ioka T., Ando H., Ito C., Akimoto T., Fujimura A., Asano Y., Kusano E. (2006). Indoxyl sulfate stimulates proliferation of rat vascular smooth muscle cells. Kidney Int..

[12] Barreto F.C., Barreto D.V., Liabeuf S., Meert N., Glorieux G., Temmar M., Choukroun G., Vanholder R., Massy Z.A., European Uremic Toxin Work Group (EUTox) (2009). Serum indoxyl sulfate is associated with vascular disease and mortality in chronic kidney disease patients. Clin. J. Am. Soc. Nephrol..

[13] Lin C.J., Liu H.L., Pan C.F., Chuang C.K., Jayakumar T., Wang T.J., Chen H.H., Wu C.J. (2012). Indoxyl sulfate predicts cardiovascular disease and renal function deterioration in advanced chronic kidney disease. Arch. Med. Res..

[14] Cao X.S., Chen J., Zou J.Z., Zhong Y.H., Teng J., Ji J., Chen Z.W., Liu Z.H., Shen B., Nie Y.X. (2015). Association of indoxyl sulfate with heart failure among patients on hemodialysis. Clin. J. Am. Soc. Nephrol..

[15] Angoff R., Mosarla R.C., Tsao C.W. (2021). Aortic stiffness: Epidemiology, risk factors, and relevant biomarkers. Front. Cardiovasc. Med..

[16] Herzog M.J., Müller P., Lechner K., Stiebler M., Arndt P., Kunz M., Ahrens D., Schmeißer A., Schreiber S., Braun-Dullaeus R.C. (2025). Arterial stiffness and vascular aging: Mechanisms, prevention, and therapy. Signal Transduct. Target. Ther..

[17] Benetos A., Laurent S., Hoeks A.P., Boutouyrie P., Safar M.E. (1993). Arterial alterations with aging and high blood pressure: A noninvasive study of carotid and femoral arteries. Arterioscler. Thromb. J. Vasc. Biol..

[18] Castelli R., Gidaro A., Casu G., Merella P., Profili N.I., Donadoni M., Maioli M., Delitala A.P. (2023). Aging of the arterial system. Int. J. Mol. Sci..

[19] Xin Y., Zhang Z., Lv S., Xu S., Liu A., Li H., Li P., Han H., Liu Y. (2024). Elucidating VSMC phenotypic transition mechanisms to bridge insights into cardiovascular disease implications. Front. Cardiovasc. Med..

[20] Schram M.T., Henry R.M., van Dijk R.A., Kostense P.J., Dekker J.M., Nijpels G., Heine R.J., Bouter L.M., Westerhof N., Stehouwer C.D. (2004). Increased central artery stiffness in impaired glucose metabolism and type 2 diabetes: The Hoorn Study. Hypertension.

[21] Staef M., Ott C., Kannenkeril D., Striepe K., Schiffer M., Schmieder R.E., Bosch A. (2023). Determinants of arterial stiffness in patients with type 2 diabetes mellitus: A cross sectional analysis. Sci. Rep..

[22] Lin C.N., Wu I.W., Huang Y.F., Peng S.Y., Huang Y.C., Ning H.C. (2019). Measuring serum total and free indoxyl sulfate and p-cresyl sulfate in chronic kidney disease using UPLC-MS/MS. J. Food Drug Anal..

[23] Huang S.T., Shu K.H., Cheng C.H., Wu M.J., Yu T.M., Chuang Y.W., Chen C.H. (2012). Serum p-cresol and indoxyl sulfate levels predict progression of chronic kidney disease in renal transplant recipients. Transplant. Proc..

[24] Lano G., Burtey S., Sallée M. (2020). Indoxyl Sulfate, a uremic endotheliotoxin. Toxins.

[25] Hobson S., Arefin S., Rahman A., Hernandez L., Ebert T., de Loor H., Evenepoel P., Stenvinkel P., Kublickiene K. (2023). Indoxyl sulphate retention is associated with microvascular endothelial dysfunction after kidney transplantation. Int. J. Mol. Sci..

[26] Matsumoto T., Takayanagi K., Kojima M., Katome T., Taguchi K., Kobayashi T. (2019). Direct impairment of the endothelial function by acute indoxyl sulfate through declined nitric oxide and not endothelium-derived hyperpolarizing factor or vasodilator prostaglandins in the rat superior mesenteric artery. Biol. Pharm. Bull..

[27] Liabeuf S., Desjardins L., Massy Z.A., Brazier F., Westeel P.F., Mazouz H., Titeca-Beauport D., Diouf M., Glorieux G., Vanholder R. (2016). Levels of indoxyl sulfate in kidney transplant patients, and the relationship with hard outcomes. Circ. J..

[28] Yang H.H., Chen Y.C., Ho C.C., Hsu B.G. (2024). Serum phenylacetylglutamine among potential risk factors for arterial stiffness measuring by carotid-femoral pulse wave velocity in patients with kidney transplantation. Toxins.

[29] Chiu L.T., Lin L., Lin H.J., Lai Y.H., Hsu B.G. (2022). Positive correlation of serum indoxyl sulfate level with peripheral arterial disease in hemodialysis patients. Vascular.

[30] Williams B., Mancia G., Spiering W., Agabiti Rosei E., Azizi M., Burnier M., Clement D.L., Coca A., de Simone G., Dominiczak A. (2018). 2018 ESC/ESH Guidelines for the management of arterial hypertension. Eur. Heart J..

